# Heart rate variability changes during high frequency yoga breathing and breath awareness

**DOI:** 10.1186/1751-0759-5-4

**Published:** 2011-04-13

**Authors:** Shirley Telles, Nilkamal Singh, Acharya Balkrishna

**Affiliations:** 1 Patanjali Research Foundation, Patanjali Yogpeeth, Haridwar, India

## Abstract

**Background:**

Pre and post comparison after one minute of high frequency yoga breathing (HFYB) suggested that the HFYB modifies the autonomic status by increasing sympathetic modulation, but its effect during the practice was not assessed.

**Methods:**

Thirty-eight male volunteers with group average age ± S.D., 23.3 ± 4.4 years were each assessed on two separate days in two sessions, (i) HFYB and (ii) breath awareness. Each session was for 35 minutes, with 3 periods, i.e., pre (5 minutes), during HFYB or breath awareness (15 minutes) and post (5 minutes).

**Results:**

There was a significant decrease in NN50, pNN50 and the mean RR interval during and after HFYB and after breath awareness, compared to the respective 'pre' values (*p *< 0.05) (repeated measures ANOVA followed by *post-hoc *analysis). The LF power increased and HF power decreased during and after breath awareness and LF/HF ratio increased after breath awareness (*p *< 0.05).

**Conclusion:**

The results suggest that there was reduced parasympathetic modulation during and after HFYB and increased sympathetic modulation with reduced parasympathetic modulation during and after breath awareness.

## Background

Yoga practice places particular emphasis on voluntary breath regulation, as this is traditionally believed to influence brain functions [1]. In yoga practice there are several ways in which a practitioner may voluntarily modify their respiration, which includes changing the rate, depth, and breathing through the mouth, among other methods [2].

Modifying the rate of breathing is the basis for a particular technique called *kapalabhati*, which is a high frequency yoga breathing technique (with a breath rate of 1.0 to 2.0 Hz) [3].

This high frequency yoga breathing (HFYB) technique could be expected to alter cardiac functioning, and indeed this was seen in a study on twenty-four volunteers who practiced the technique at the rate of 2.0 Hz for 15 minutes, analyzed as three, five minute periods [4]. A 0.1 Hz rhythm was present in the record of R-R intervals and the blood pressure during the HFYB. The results were taken to support the hypothesis about the integrative role of cardiovascular and respiratory rhythms when the respiratory frequency is altered. The same authors further assessed the effects of the HFYB (at 2.0 Hz) as three, five minute periods, as in their other study, in seventeen advanced yoga practitioners [5]. The EKG, respiration and blood pressure were recorded continuously during the three, five minute periods of HFYB, as well as before and after the practice. The beat to beat series of the R-R intervals as well as systolic and diastolic blood pressure were analyzed by a spectral analysis of the time series. All frequency bands of R-R interval variability were reduced in HFYB. These results as well as the changes in other variables point to decreased cardiac vagal modulation during HFYB. The changes in R-R interval variability, as well as in the blood pressure, were interpreted by the authors as suggestive of decreased cardiac vagal modulation during HFYB.

A comparable result was reported when twelve experienced practitioners practiced the HFYB technique at 2.0 Hz for one minute and a frequency domain analysis was carried out [6]. Following one minute of HFYB there was a significant increase in the low frequency power and in the LF/HF ratio, while the HF power was significantly lower following the HFYB. The results suggested that the HFYB modifies the autonomic status by increasing sympathetic activity with reduced vagal activity.

The effect of HFYB on the cardiac autonomic control was hence reported to have comparable effects in two studies which differed in the duration of the practice i.e., fifteen minutes [5] as compared to one minute [6].

The same HFYB practice was shown to improve the performance in a cancellation task requiring both focused and selective attention [7]. The participants here were forty-six medical students, forty-eight middle-aged adults (30 to 59 years), and sixteen older adults (all of whom were over 60 years of age). The HFYB was practiced at rates varying between 1.0 and 2.0 Hz, for 15 minutes.

Apart from the performance in a paper and pencil cancellation task [7], the HFYB at 2.0 Hz for one minute also improved the performance in a P300 event related potential task [8]. The P300 is an event related potential which is generated when attending to and differentiating between auditory stimuli [9]. The P300 was recorded in two groups with fifteen participants in each group. One group practiced the HFYB at 2.0 Hz for one minute, while the other group practiced being aware of their breath, for the same duration of time. The P300 changed after both practices. A decreased latency of the P300 after the HFYB suggested a decrease in the time required to complete this attentional task. In contrast following breath awareness an increase in the P300 amplitude which was recorded, suggested that greater neural resources were needed to complete the task.

Attention and vigilance are associated with increased sympathetic modulation [10]. Since HFYB (at 1.0 Hz and at 2.0 Hz) improved the performance in an attentional task [7,8], the present study was undertaken to assess the effect of fifteen minutes (as three, five minute epochs) of the HFYB on heart rate variability with both time and frequency domain analysis, in a larger sample than was previously studied, i.e., in thirty-eight participants. All thirty-eight participants were assessed in two sessions. These were before, during, and after (i) HFYB and (ii) a comparable duration of breath awareness. Breath awareness was selected as the second technique practiced for two reasons, i.e., (i) breath awareness is believed to improve attention [7] and hence may influence sympathetic modulation and (ii) breath awareness is a part of all yoga breathing techniques, including the HFYB (or *kapalabhati*) studied here.

## Methods

### Subjects

There were 38 male volunteers with ages ranging between 17 and 35 years (group mean ± S.D., 23.3 ± 4.4 years). Based on a routine clinical examination all the participants were found to be in normal health and none of them were taking any medication. They were all living in the north of India and working as security personnel. All of them ate a vegetarian diet and their experience of yoga breathing practices ranged between 3 and 84 months. Their practice of the two techniques was observed and verified to be correct by an experienced yoga instructor.

### Design

Every participant was assessed in two types of practice sessions (high frequency yoga breathing or *kapalabhati *and breath awareness) and assessments were made on two different days for every participant. The sequence of practice was reversed for alternate participants. The study design was explained to the participants and their signed informed consent was obtained. The study had the approval of the institution's ethics committee.

### Assessments

The participants stayed in the research laboratory one night prior to the day of recording and they were requested to avoid physical exertion. The actual recording was done the next morning between 05:00 and 07:30 hours. These precautions were taken so that their basal autonomic status could be assessed, before they had any physical or mental activity other than the two yoga techniques. In most cases the practices were assessed on the next day at the same time of the day. In those cases where the assessments were not on consecutive days the interval between practices was at least 48 hours and did not exceed 3 days. The data were collected between March and August 2010.

The EKG and respiration were assessed throughout a session which lasted for 35 minutes (with three minutes for rest in between), as described below. During the session the participants practiced either *high frequency yoga breathing *(HFYB) or breath awareness for 15 minutes interspersed with 1 minute of rest after every 5 minutes of practice. Hence the 15 minutes was divided into three epochs of 5 minutes each. The practice period was preceded by 5 minutes and followed by 15 minutes, during which participants breathed normally. These were the 'pre' and 'post' periods, respectively.

**Figure 1:**
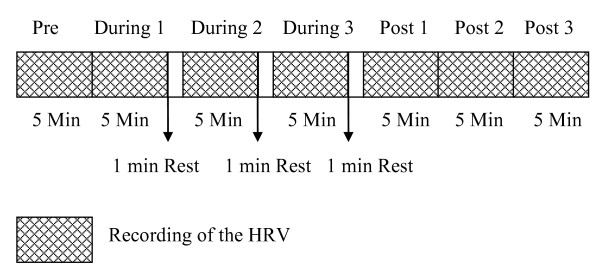
**Design of the HFYB and breath awareness sessions showing the periods during which the HRV was recorded**.

Throughout the session participants were seated crossed legged keeping their spine straight. The total duration of each session was 38 minutes, i.e., 5 minutes before the practice, 18 minutes during the practice, and 15 minutes after the practice. Assessments were taken continuously in the pre, during 1, during 2, during 3, post 1, post 2 and post 3 periods of 5 minutes each as shown in Figure 1. No recordings were made during three, 1-minute rest periods. This has been schematically presented in Figure 1.

Heart rate variability was assessed using a two channel EKG and respiration recording system (MP 45 Biopac Student Lab, BIOPAC System Inc, U.S.A.). The EKG was recorded using Ag/AgCl pre-gelled electrodes (Tyco Healthcare, Germany) and recording was made with a standard limb lead I configuration. Data were acquired at the sampling rate of 1024 Hz and were analyzed offline. Noise free data were included for analysis.

The respiratory rate was recorded using an open circuit oxygen consumption analyzer (Quark CPET, Italy).

### Data extraction

Recordings of the heart rate variability and heart rate were taken for 38 minutes for each participant. The pre-intervention (5 minutes), intervention (15 minutes as the 3 minutes of rest were not included) and post-intervention (15 minutes) data were analyzed separately. The HRV power spectrum was obtained using Fast Fourier Transform (FFT) analysis.

The energy in the HRV series in two specific frequency bands was studied viz., low frequency (LF) band (0.05-0.15 Hz) and high frequency (HF) band (0.15-1.50 Hz). The high frequency band used in the present study for data extraction for both HFYB and breath awareness sessions is not the conventional band, i.e., 0.15-0.40 Hz. The change to 0.15 - 1.50 Hz was made considering that the breathing frequency during HFYB was approximately 1.0 Hz. The LF/HF ratio was also calculated. The low frequency and high frequency band values were expressed as normalized units. In addition to frequency domain analysis, time domain analysis was also done. The following components of time domain HRV were analyzed: (i) mean RR interval (the mean of the intervals between adjacent QRS complexes or the instantaneous heart rate), (ii) NN50 (the number of interval differences of successive NN intervals greater than 50 ms), and (iii) pNN50 (the proportion derived by dividing NN50 by the total number of NN intervals. Mean respiratory rate was calculated pre, during and post sessions.

### Data analysis

Repeated measures analyses of variance (ANOVA) followed by *post-hoc *analyses with Bonferroni adjustment were done to compare data recorded during and after the two practices with data recorded before the two practices, using SPSS Version 18.0. There were two Within subjects factors. These were Sessions (HFYB and Breath awareness) and States (Pre, During and Post).

## Results

### Repeated measures analyses of variance (RMANOVA)

#### Heart Rate Variability

*1) Time domain analysis*. The mean RR interval showed a significant difference between (i) Sessions [F = 47.11, df = 1, 37.0, *p *< .001 (*p *= 4.31 E-08)], (ii) States [F = 22.3, df = 2.81, 104.08, Huynh-Feldt epsilon = 0.469, *p *< .001 (*p *= 8.42 E-11)] and (iii) Sessions × States [F = 59.38, df = 2.55, 93.82, Huynh-Feldt epsilon = 0.423, *p *< .001 (*p *= 8.6 E-20)]. For the NN50 there was a significant difference between (i) Sessions [F = 30.84, df = 1, 37, *p *< .001 (*p *= 2.53 E-06)], (ii) States [F = 21.32, df = 1.84, 68.05, Huynh-Feldt epsilon = 0.307, *p *< .001 (*p *= 1.46 E-07)] and (iii) the interaction between Sessions × States [F = 37.15, df = 1.40, 51.68, Huynh-Feldt epsilon = 0.233, *p *< .001 (*p *= 5.53 E-09)]. The pNN50 showed a significant difference between (i) Sessions [F = 39.60, df = 1, 37, *p *< .001 (*p *= 2.52 E-07)], (ii) States [F = 25.01, df = 2.09, 77.45, Huynh-Feldt epsilon = 0.349, *p *< .001 (*p *= 2.41 E-09)] and (iii) Sessions × States [F = 46.19, df = 1.47, 54.34, Huynh-Feldt epsilon = 0.245, *p *< .001 (*p *= 1.07 E-10)].

*2) Frequency domain analysis*. There were no significant difference between sessions, states or their interaction for LF, HF, and LF/HF.

#### Respiratory Rate

There was a significant difference between Sessions [F = 1264, df = 1, 37, *p *< .001 (*p *= 3.26 E-30)], States [F = 918.55, df = 2.23, 82.34, Huynh-Feldt epsilon = 0.371, *p *< .001 (*p *= 1.18 E-58)] and interaction between Sessions × States [F = 1000.30, df = 2.01, 74.44, Huynh-Feldt epsilon = 0.335, *p *< .001 (*p *= 1.34 E-54)].

### *Post - hoc *analyses

#### Heart rate variability

*1) Time domain analysis*. During the HFYB session a significant decrease was observed in mean RR interval, NN50 and pNN50 when compared with pre-intervention (*p *< .001), there was also a significant decrease in mean RR interval when post-intervention was compared with pre (*p *< .01), and in pNN50 when post-intervention was compared with pre (*p *< .05).

In the breath awareness session there was a significant reduction post-intervention compared to pre for the mean RR interval (*p *< .05), NN50 (*p *< .01) and the pNN50 (*p *< .01).

*2) Frequency domain analysis*. During breath awareness there was a significant increase in LF compared with pre-intervention (*p *< .05) and when post-intervention was compared with pre-intervention (*p *< .05). A significant decrease was observed in HF during-intervention compared with pre-intervention (*p *< .05); and when post-intervention was compared with pre-intervention (*p *< .05). There was a significant increase in LF/HF ratio when post-intervention was compared with pre-intervention (*p *< .01).

#### Respiratory rate

During the HFYB session there was an expected significant increase in respiratory rate compared with pre-intervention (*p *< .001).

**Figure 2:**
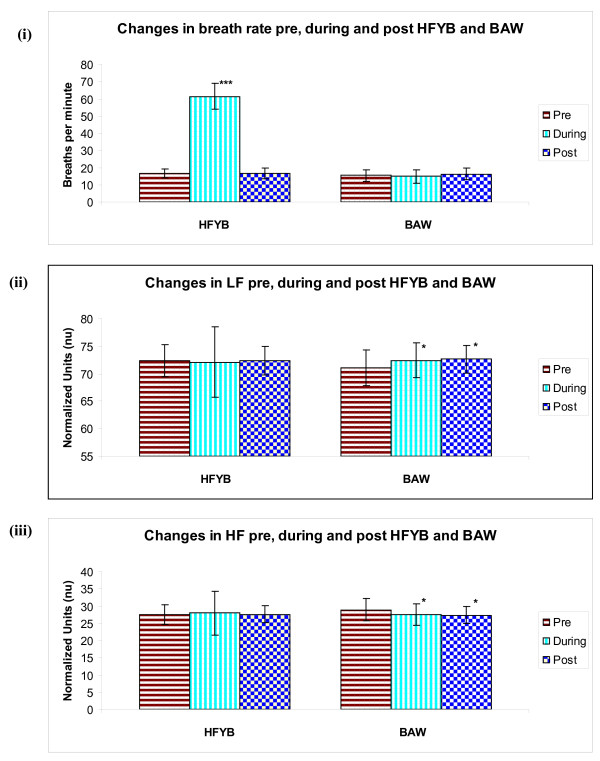
**Changes in (i) breath rate, (ii) LF and (iii) HF, Pre, During and Post HFYB and breath awareness**.

**Figure 3:**
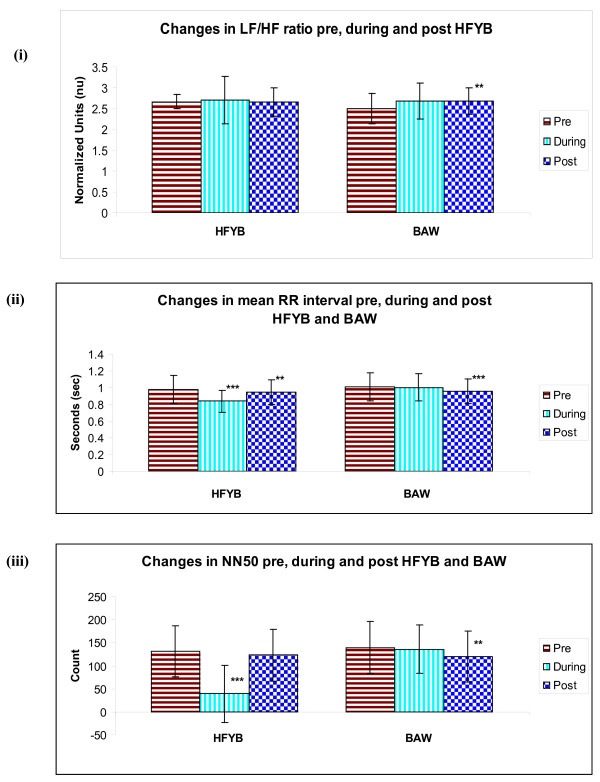
**Changes in (i) LF/HF, (ii) mean R-R interval and (iii) NN50, Pre, During and Post HFYB and breath awareness**.

**Figure 4:**
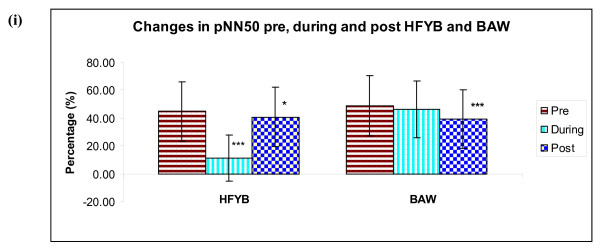
**Changes in (i) pNN50, Pre, During and Post HFYB and breath awareness**.

The mean values ± S.D. of pre, during and post-intervention for both HFYB and breath awareness sessions for respiratory rate, LF, HF, LF/HF ratio, mean RR interval, NN50, and pNN50 are given in Table 1 and Table 2, and in Figure 2, Figure 3 and Figure 4.

**Table 1:** Breath rate and HRV measures in HFYB sessions.

Variables	Pre	During 1	During 2	During 3	Post 1	Post 2	Post 3
Breath rate (breaths/min)	16.45 ± 2.61	60.64 ± 7.39***	61.13 ± 7.24***	62.54 ± 7.58***	16.02 ± 2.87	16.66 ± 3.09	16.55 ± 3.30
LF (nu)	72.37 ± 2.86	71.80 ± 6.68	71.85 ± 6.57	72.52 ± 6.00	71.72 ± 2.67	72.36 ± 2.43	73.05 ± 2.74
HF (nu)	27.63 ± 2.86	28.20 ± 6.68	28.15 ± 6.57	27.48 ± 6.00	28.28 ± 2.67	27.64 ± 2.43	26.95 ± 2.74
LF/HF (nu)	2.67 ± 0.53	2.67 ± 0.57	2.68 ± 0.59	2.75 ± 0.54	2.57 ± 0.32	2.64 ± 0.31	2.75 ± 0.40
Mean RR (sec)	0.98 ± 0.17	0.84 ± 0.14***	0.84 ± 0.14***	0.84 ± 0.12***	0.96 ± 0.15	0.96 ± 0.15	0.93 ± 0.14**
NN50 (count)	132.03 ± 55.02	40.76 ± 60.03***	38.16 ± 65.65***	40.00 ± 60.14***	129.03 ± 56.43	126.58 ± 56.29	115.63 ± 56.24
pNN50 (%)	44.76 ± 21.51	11.39 ± 15.31***	10.55 ± 16.79***	11.53 ± 16.92***	42.77 ± 21.49	41.79 ± 21.50	37.40 ± 21.15*

*p < 0.05, **p < 0.01 and ***p < 0.001, Repeated measures analyses of Variance (RMANOVA), with *post-hoc *analyses comparing During and Post

**Table 2:** Breath rate and HRV measures in breath awareness sessions.

Variables	Pre	During 1	During 2	During 3	Post 1	Post 2	Post 3
Breath rate (breaths/min)	15.39 ± 3.52	14.61 ± 3.80	15.03 ± 3.79	14.93 ± 3.76	16.13 ± 3.32	16.24 ± 3.33	16.20 ± 3.74
LF (nu)	71.09 ± 3.30	72.08 ± 3.43	72.52 ± 3.50	72.63 ± 2.62*	72.39 ± 2.80	72.73 ± 2.21*	72.81 ± 2.49*
HF (nu)	28.91 ± 3.30	27.92 ± 3.43	27.48 ± 3.50	27.37 ± 2.62*	27.61 ± 2.80	27.27 ± 2.21*	27.19 ± 2.49*
LF/HF (nu)	2.50 ± 0.36	2.63 ± 0.43	2.70 ± 0.48	2.69 ± 0.38	2.66 ± 0.35	2.69 ± 0.30	2.71 ± 0.32**
Mean RR (sec)	1.01 ± 0.17	1.01 ± 0.16	1.00 ± 0.16	1.00 ± 0.16	0.98 ± 0.15*	0.95 ± 0.15***	0.94 ± 0.15***
NN50 (count)	139.95 ± 55.77	137.97 ± 51.33	134.87 ± 54.23	133.53 ± 51.92	127.00 ± 51.98	122.03 ± 53.62	110.21 ± 59.33**
pNN50 (%)	48.69 ± 21.62	47.09 ± 19.85	46.07 ± 21.05	45.45 ± 20.61	42.74 ± 20.34	39.31 ± 20.57**	35.69 ± 21.71***

*p < 0.05, **p < 0.01 and ***p < 0.001, Repeated measures analyses of Variance (RMANOVA), with *post-hoc *analyses comparing During and Post

## Discussion

Time domain analyses of both HFYB and breath awareness were associated with changes in the heart rate variability during and after the practice which were suggestive of reduced parasympathetic modulation. The magnitude of change during the practice was more during HFYB whereas the magnitude of change after the practice was higher after breath awareness. Frequency domain analysis showed changes during and after breath awareness suggestive of increased sympathetic and decreased parasympathetic modulation.

The time domain measures (i.e., the mean R-R interval, NN50 and pNN50) are recognized to be strongly dependent on the vagal modulation [11,12]. The decrease in the mean RR interval, NN50 and pNN50 during and after HFYB were significant whereas the changes were significant only after breath awareness but not during the practice. Hence the decrease in vagal modulation occurred during and after HFYB and after breath awareness but not during breath awareness. The decrease in vagal modulation described above for HFYB and breath awareness could be related to the fact that these practices are associated with increased vigilance and better performance in an attention task [7]. This is relevant since attention and vigilance are associated with higher sympathetic modulation in human volunteers and animal models [13,14].

There were no changes in variables obtained by frequency domain analysis during or after HFYB. In contrast the LF significantly increased during and after breath awareness whereas the HF decreased during and after breath awareness. The LF band of the heart rate variability is mainly related to sympathetic activation when expressed in normalized units [12], while efferent vagal activity chiefly contributes to the HF band [15].

The absence of change in the frequency domain analysis variables during HFYB could suggest that HFYB was associated with parasympathetic withdrawal but not with simultaneous increase in sympathetic modulation. In the present study it is also essential to remember that the HF band was extended from the conventional 0.15 - 0.40 Hz to 0.15 - 1.50 Hz. This was done to match the breathing frequency during HFYB where breathing frequency was approximately 1.0 Hz. While the breathing frequency was not modified for breath awareness, the band was extended for analysis of heart rate variability during breath awareness as well. This change may have influenced the results.

Concurrent monitoring of respiration and the heart rate variability are considered important. This has been supported by an acute increase in LF and total spectrum heart rate variability and in vagal baroreflex gain corrected with slow breathing during biofeedback [16]. Biofeedback training used to increase the amplitude of respiratory sinus arrhythmia maximally increases the amplitude of heart rate oscillations at approximately 0.1 Hz [17]. In the present study during HFYB the respiratory frequency was maintained at approximately 1.0 Hz, however there was no increase in HF power during HFYB, hence the changes observed in the present study appear to be related to changes in autonomic modulation rather than being a result of the change in respiratory rate.

The study has two main limitations. (i) It would have been ideal to test the subjects after a time interval with no intervention as the study included two yoga practices, but no control. (ii) In the present study the breath frequency was close to the frequency of the heart rate during HFYB. This could have resulted in interference between two close frequencies and given rise to artifact, particularly increasing the LF (which was not seen).

In summary both HFYB and breath awareness were associated with a shift in the autonomic balance towards vagal withdrawal. This was greater during HFYB and after breath awareness. Breath awareness was also associated with changes suggestive of increased sympathetic modulation during and after the practice. As mentioned earlier these results support earlier findings [7] that both practices increase performance in an attention task since increased attention is associated with higher sympathetic modulation. While the sympathetic modulation was not increased during HFYB, the vagal withdrawal during HFYB could result in a shift in autonomic balance towards sympathetic dominance. The exact mechanism by which both practices may be influencing the autonomic nervous system remains a speculation. The respiratory and cardiovascular centers are closely associated in the brainstem. It is possible that conscious cortical regulation of brainstem respiratory centers may influence cardiovascular centers and hence bring about the changes in heart rate variability seen. However at this stage this is merely a speculation.

## Conclusion

Both HFYB and breath awareness resulted in decreased parasympathetic modulation. These changes remained for 15 minutes after the practice. Breath awareness was also associated with increased sympathetic modulation.

## Competing interests

The authors declare that they have no competing interests.

## Authors' contributions

ST designed the study, interpreted the data and compiled the manuscript. NS collected the data, performed statistical analysis and helped in compiling the manuscript. AB decided the details of the intervention. All authors (ST, NS and AB) read and approved the final version of the manuscript.
